# Editorial: Neglected lung diseases in the COVID-19 era

**DOI:** 10.3389/fmed.2022.1016008

**Published:** 2022-09-21

**Authors:** Fatma Yildirim, Sevket Özkaya, Özlem Erçen Diken, Christophe von Garnier

**Affiliations:** ^1^Department of Pulmonary Medicine, Pulmonary Critical Care Unit, University of Health Sciences, Dişkapi Yildirim Beyazit Research and Education Hospital, Ankara, Turkey; ^2^Department of Pulmonary Medicine, Faculty of Medicine, Bahçeşehir University, Istanbul, Turkey; ^3^Altinbaş University Faculty of Medicine, Department of Pulmonary Medicine, Istanbul, Turkey; ^4^Department of Pulmonary Medicine, University of Health Sciences, Adana City Hospital, Adana, Turkey; ^5^Division of Pulmonology, Department of Medicine, Lausanne University Hospital (CHUV), University of Lausanne, Lausanne, Switzerland

**Keywords:** COVID-19, exacerbation of lung parenchymal diseases, confusion of diagnosis of pulmonary involvements, increased burden on the healthcare system due to COVID-19, pandemic (COVID-19)

The coronavirus disease 2019 (COVID-19), caused by Severe Acute Respiratory Syndrome Coronavirus-2 (SARS-CoV-2), has turned into a major pandemic all over the world and has been going on for 2.5 years (1). Since SARS-CoV-2 primarily affects the respiratory system, patients with pre-existing parenchymal lung disease were more affected during the pandemic. In this direction, Goto et al. reported a case with SARS-CoV-2 triggered acute exacerbation of idiopathic pulmonary fibrosis (IPF) in a patient with pre-existing IPF in our topic.

After recovering from COVID-19, the majority of patients suffered from deterioration in pulmonary function after they were discharged from the hospital. In this sense, pulmonary rehabilitation of these patients in the post-COVID period is crucial. In the meta-analysis they conducted on this topic, Chen et al. showed that pulmonary rehabilitation provided an increase in the 6-minute walking test, an improvement in the symptoms of dyspnea, anxiety, and depression scores, and an increase in quality of life in patients who had COVID-19 with lung involvement. However, the proper time to initiate pulmonary rehabilitation after COVID-19 has been a matter of debate since the beginning of the pandemic. In their meta-analysis, the mean time to start pulmonary rehabilitation in hospitalized patients was 70.1 ± 16.9 days. In other studies from the literature, it has been observed that pulmonary rehabilitation was started as soon as weaning is performed from the mechanical ventilator in the intensive care unit. Due to the heterogeneity of the studies, no inference could be made for the degree of change in pulmonary function tests in this meta-analysis.

During the COVID-19 pandemic, an exacerbation of pre-existing pulmonary disease and the diagnosis of COVID-19 could not be differentiated due to the similarities in patients' symptoms and chest computed tomography (CT) findings. In their case reported in this section, Torun and Karaman reported an exacerbation of pulmonary involvement of rheumatoid arthritis (RA) which was confused with COVID-19. In this case, the patient was evaluated as a possible COVID-19 case due to bilateral and central ground-glass opacities in thorax CT and received specific treatment for COVID-19 twice. They reported that the patient, who had two separate negative COVID-19 real-time polymerase chain reaction (RT-PCR) results, did not improve after 3 months. After further examination, they found that the patient who had a previous diagnosis of RA was unable to go to her routine physician appointments due to the pandemic circumstances, and she stopped her RA-specific drugs on her own. This patient was then evaluated as an acute exacerbation of pulmonary involvement of RA, and she was started on cyclophosphamide treatment and improved significantly.

The COVID-19 pandemic has created a significant burden on health systems in many countries, and caused significant disruptions in the follow-up of non-COVID-19 patients due to social isolation rules, especially during peak periods. In their survey conducted by Karaman et al. from Turkey on healthcare workers, 42% of the participants stated that their workload significantly increased during the pandemic; 54.6% of them stated that they introduced the telemedicine application, which they had not used before, into their clinical practice. Especially during the peak periods of the pandemic, more than half of the participants stated that they saw only a few non-COVID-19 patients. In addition, most of the participants stated that patients with chronic diseases delayed their follow-up appointments. As can be understood from this survey, the diagnosis of many diseases in the pandemic may have been missed because physicians concentrated on COVID-19 and its complications.

SARS-CoV-2 affects many organ systems, including the diaphragm. Boussuges et al. investigated the frequency and risk factors of diaphragmatic dysfunction (DD) in patients who had severe COVID-19 infection [hospitalized with oxygen support or mechanical ventilation (MV)] and recovered. Pulmonary function tests (PFT) and diaphragmatic ultrasonography were performed on the patients in the 3rd and 4th months after discharge from the hospital. In the study in which a total of 132 patients were evaluated, 58 (44%) patients were hospitalized in the intensive care unit and MV was applied to 32 (25%) patients. It was observed that 77% of patients had persistent respiratory difficulties; a restrictive pattern in PFT was detected in 29% of patients, and a decrease in the diffusion capacity was detected in 11.5% of them. Abnormal diaphragmatic function was detected in 13 (10%) patients. While 2 of them had hemidiaphragmatic paralysis, 11 patients had DD. Patients with DD had a higher rate of a history of cardio-thoracic invasive procedure or a history of upper abdominal surgery. In logistic regression analysis, Modified Medical Research Council (mMRC) and total lung capacity in PFT were correlated with DD. This study suggests that a severe course of COVID-19 may promote preexisting DD in patients with pre-existing risk factors for DD. The absence of risk factors for 7 DD patients in this study suggests that DD may develop in patients with severe COVID-19, and patients with long-term respiratory symptoms should be evaluated for DD.

Today, COVID-19 can be considered an important cause of acute exacerbation in patients with chronic pulmonary disease. Since thorax CT findings of COVID-19 are similar to many interstitial lung diseases, other differential diagnoses of COVID-19 should not be forgotten, especially in patients with negative RT-PCR for COVID-19. In patients with COVID-19, referral to pulmonary rehabilitation after negative RT-PCR results seem to be in favor of patients. The delay in the follow-up of chronic diseases and the diagnosis of diseases such as cancer in advanced stages are the negative factors that will increase the burden on health systems and decrease patient survival in the post-pandemic period.

## Author contributions

FY planned the editorial. SÖ identified references. ÖE and CV performed revision of the text. All authors contributed to the article and approved the submitted version.

## Conflict of interest

The authors declare that the research was conducted in the absence of any commercial or financial relationships that could be construed as a potential conflict of interest.

## Publisher's note

All claims expressed in this article are solely those of the authors and do not necessarily represent those of their affiliated organizations, or those of the publisher, the editors and the reviewers. Any product that may be evaluated in this article, or claim that may be made by its manufacturer, is not guaranteed or endorsed by the publisher.
